# S100A6 (Calcyclin) is a prostate basal cell marker absent in prostate cancer and its precursors

**DOI:** 10.1038/sj.bjc.6602034

**Published:** 2004-07-27

**Authors:** I Rehman, S S Cross, A-R Azzouzi, J W F Catto, J C Deloulme, S Larre, J Champigneuille, G Fromont, O Cussenot, F C Hamdy

**Affiliations:** 1 Academic Urology Unit, Division of Clinical Sciences South, University of Sheffield, Floor K, Royal Hallamshire Hospital, Glossop Road, Sheffield S10 2JF, UK; 2 Academic Unit of Pathology, Division of Genomic Medicine, University of Sheffield, Sheffield, UK; 3 CeRePP-EA3104, Départements d'Urologie (AP-HP Tenon & IMM), Universités Paris 6 & 7, France; 4 Departement de Biologie Moleculaire et Structurale du Commissariat a l'Energie Atomique, INSERM Unite 244, Commissariat a l'Energie Atomique-Grenoble, France; 5 Service d'Anatomie Pathologique, CHU Nancy-Brabois, France

**Keywords:** S100, adenocarcinoma, prostatic intraepithelial neoplasia, cytokeratin 5, cytokeratin 18, methylation

## Abstract

S100A6 (Calcyclin) is a calcium-binding protein that has been implicated in a variety of biological functions as well as tumorigenesis. The aim of our study was to investigate the involvement of S100A6 during prostate cancer development and progression. Using immunohistochemistry, the expression of S100A6 was examined in benign (*n*=66), premalignant (*n*=10), malignant (*n*=66) and metastatic prostate (*n*=5) tissues arranged in a tissue-microarray or whole sections as well as in prostate cancer cell lines. The S100A6 immunostaining pattern in tissues was compared with that of cytokeratin 5 (a basal cell marker) and 18 (a benign luminal cell marker). In all cases of benign epithelium, intense S100A6 expression was seen in the basal cell layer with absent staining in luminal cells. In all cases of prostatic adenocarcinoma (matched), metastatic lesions and 3/10 high-grade prostatic intraepithelial neoplasia lesions, an absence of S100A6 was seen. Western blotting and RT–PCR analysis of cell lines showed S100A6 expression to be absent in LNCaP, LNCaP-LN3 and LNCaP-Pro5 but present in Du145, PC3, PC-3M and PC-3M-LN4. LNCaP cells treated with 5-Azacytidine, caused re-expression of S100A6 mRNA. Sequencing of bisulphite modified DNA showed CpG methylation within the S100A6 promoter region and exon 1 of LNCaP, LNCaP-LN3 and LNCaP-Pro5 cell lines but not in Du145 cells. Our data suggest that loss of S100A6 protein expression is common in prostate cancer development and may occur at an early stage. The mechanism of loss of expression may involve hypermethylation of CpG sites. The finding of intense S100A6 expression in the basal cells of benign glands but loss of expression in cancer could be useful as a novel diagnostic marker for prostate cancer.

Prostate cancer is among the most commonly diagnosed cancers in men and has become the second most common cause of cancer-related deaths (Greenlee *et al*, 2000; Gronberg, 2003). However, our understanding of its aetiology is limited. Molecular genetic studies have shown that the frequency of genetic alterations is low compared with other common cancers. The most common alterations identified are loss of Glutathione *S* transferase-pi (GST-pi) and Annexin expression (Lee *et al*, 1994; Paweletz *et al*, 2000; Chetcuti *et al*, 2001). The frequencies of loss of expression of GST-pi, Annexin I and II approach 100% in prostate cancer.

Calcium plays an important role in a number of physiological processes, thus intracellular calcium levels must be carefully regulated. There are three main families of calcium-binding proteins, which are thought to regulate calcium levels (Schafer and Heizmann, 1996; Hawkins *et al*, 2000; Michalak *et al*, 2002; Donato, 2003). The first family includes the endoplasmic reticulum calcium-binding proteins; the second family includes the Annexins and the third family includes the S100 members. Members of the S100 protein family have been implicated in a variety of cellular activities such as protein phosphorylation, enzyme activities, cell proliferation and differentiation, as well as in tumorigenesis (Schafer and Heizmann, 1996; Heizmann *et al*, 2002; Donato, 2003). It has also been reported that proteins from two different families may form heterodimer complexes, such as the interaction between S100A6 and Annexin II and between S100B and S100A6 (Zeng *et al*, 1993, Kuo *et al*, 1997; Deloulme *et al*, 2000).

S100A6 was initially identified as an mRNA preferentially expressed in proliferating rather than quiescent cells (Calabretta *et al*, 1986). Subsequent studies showed that increased S100A6 expression is associated with the metastatic potential of human melanoma cell lines and primary melanomas (Weterman *et al*, 1992, 1993). Protein expression has been found to be increased at the invading fronts of colorectal adenocarcinomas, and may be involved in the invasion process (Komatsu *et al*, 2000). In astrocytic tumours S100A6 protein expression correlates with tumour grade (Camby *et al*, 1999). Recently, S100A6 has been identified as a differentially expressed gene in pancreatic adenocarcinomas (Logsdon *et al*, 2003). Other than one recent report showing S100A6 expression in ∼20% of prostatic cancers arranged on a tissue-microarray (Hsieh *et al*, 2003), there are no reports investigating S100A6 expression in benign and malignant prostate tissues or cell lines. We therefore examined the tissue expression of S100A6 in benign, malignant and metastatic prostate tissues and in prostate cancer cell lines. The possible mechanism for the absence of expression in prostate cancer cell lines was investigated by bisulphite modified DNA sequencing of CpG sites within the S100A6 promoter and 5′ upstream sequence of exon 1.

## MATERIALS AND METHODS

### Tissue specimens

Following local ethics committee approval and informed consent, tissues were collected from a total of 71 patients, who attended the Urology clinic at three centres: Academic Urology Unit, University of Sheffield; CeRePP-EA3104, Départements d'Urologie, Universités Paris or Service d'Anatomie Pathologique, CHU Nancy-Brabois (France). Paraffin-embedded tissue in blocks from 28 histologically proven cases of organ confined prostate cancer obtained either by radical prostectomy or Transurethral resection of prostate (TURP) were collected at the Universités Paris. Organ confined tumours had Gleason scores ranging from 3 to 6 (*n*=3), 7 (*n*=13) and 8–10 (*n*=12). Four cases of metastatic prostate cancer in lymph node were collected at the Service d'Anatomie Pathologique and one case of metastatic prostate cancer in bone was collected at the University of Sheffield.

### Tissue microarrays

For the construction of Tissue Microarrays (TMA), 38 tumour tissue samples obtained either by radical prostectomy or TURP were collected at the University of Sheffield. Areas representing the largest carcinoma as well as areas of normal appearing epithelium and normal appearing stroma were circled on the haematoxylin & eosin stained slides. For each area, triplicate 0.6 mm cores were obtained from the circled areas and were transferred onto recipient paraffin blocks. Tumour areas sampled generally reflected the final Gleason score for the case and included low, intermediate and high-grade adenocarcinomas. In total, 5-*μ*m sections from the array blocks and tissues were cut for immunohistochemistry.

### Prostate cancer cell lines and culture

PC3, Du145 and LNCaP cell lines were obtained from the American-Type Culture Collection (Manassas, VA, USA). PC-3M, PC-3M-LN4, LNCaP-LN3 and LNCaP-Pro5 cell lines were kindly provided by Dr CA Pettaway (University of Texas MD Anderson Cancer Centre, Houston, TX, USA) (Pettaway *et al*, 1996). All cell lines were maintained in RPM1-1640 with 10% foetal calf serum, glutamine, vitamins, amino-acids and antibiotics.

### RNA extraction

RNA was extracted using TRI reagent (Sigma-Aldrich, Dorset, UK), according to the manufacturer's instructions. After precipitation the pellet was washed three times with 75% ethanol. All RNA was quantified spectrophotometrically.

### RT–PCR

RNA (2 *μ*g) was reverse transcribed into cDNA using a reverse transcription kit (Invitrogen), with 250 ng of random primers according to the manufacturer's instructions. For S100A6 PCR two primer pairs were designed using the cDNA sequence of the Ensembl genome browser (Ensembl gene i.d. ENSG00000160670). The sequence of the forward and reverse primer pairs were:

S100A6F1: GTAAACCGCGAATGTGCGTTGT (exon 1)
S100A6R1: GAGTACTTGTGGAAGATGGCCA (exon 2)
S100A6F2: AGCTGAAGGAGCTGATCCAGAA (exon 2)
S100A6R2: CCCTTGAGGGCTTCATTGTAGA (exon 3)

GAPDH PCR was performed to test RNA integrity and as a loading control. Primer sequences were

GAPDHF: GAAGGTGAAGGTCGGAGTC
GAPDHR: GAAGATGGTGATGGGATTTC

All primers were synthesised by MWG-Biotech (UK). RT–PCR was performed using 200 ng of each primer in 20 *μ*l volumes. Touch down thermocycling was performed using the following annealing temperatures, three cycles each of 65°C for 30 s, 62°C for 30 s, 59°C for 30 s then 30 cycles of 56°C for 1 min. All denaturation steps were 94°C for 30 s and all extension steps were 72°C for 30 s. PCR controls consisted of nontranscribed total RNA and water. Amplified products were separated on 2.5% agarose gels containing ethidium bromide.

### Immunohistochemistry

Immunohistochemistry was performed essentially as previously described with the following modifications (Rehman *et al*, 1996). Nonspecific sites were blocked using 1 × Casein (Vector laboratories, Peterborough, UK) in 0.5 M Tris-buffered saline (TBS), 2 mM CaCl_2_, 0.05% Tween 20, pH 7.5. Sections were incubated for 1 h with rabbit anti-S100A6 polyclonal antibody at a 1 : 1000 dilution (Cat. No. A5115, Dako, Cambs, UK) or Mouse monoclonal anti-S100A6 antibody (Cat. No. S5049, Sigma-Aldrich, Dorset, UK) at a 1 : 2000 dilution in 1/4 × Casein in 0.5 M TBS, 2 mM CaCl_2_, 0.012% Tween 20, pH 7.5. Secondary antibodies were either biotinylated Goat anti-rabbit (1 : 400 dilution) (Vector laboratories) or biotinylated rabbit anti-mouse (1 : 400 dilution) (Dako). Cytokeratin 5 and 18 immunostaining was performed using mouse monoclonal antibodies (NCL-L-CK5 and NCL-CK18, Novocastra laboratories, Newcastle, UK). S100B immunostaining was performed using the monoclonal mouse anti-S100B antibody (Cat No. M7221, Dako), at a 1 : 30 dilution. For immunostaining of cell lines, cells fixed with 4% paraformaldehdye/PBS were first treated with hot sodium citrate pH 6.0 for 15 min, and the staining performed as described above. Negative controls were included for most tissues and included omission of primary antibody. All immunostained tissues were scored by an expert histopathologist (SSC).

### Western blotting

Protein was extracted from all seven cell lines using the mammalian cell lysis kit (Sigma-Aldrich) and the NER-PER kit (Pierce Biotechnology), according to the manufacturer's instructions. Protein extracts (20 *μ*g each) were run on 12% SDS-Tris-glycine gels and blotted onto immobilon PVDF transfer membranes (Millipore, Watford, UK) for 1 h at 65 V. Blots were then blocked by 5% non-fat milk for 1 h, incubated with a 1 : 2000 dilution of the anti-S100A6 polyclonal antibody (Cat No. A5115, Dako), overnight at 4°C. After incubation with secondary peroxidase conjugated horse anti-rabbit antibody at a 1 : 30 000 dilution (Cat No. NA934 Amersham Biosciences, Bucks, UK), the membranes were developed by ECL Advanced Western blotting detection kit (Amersham Biosciences) for X-ray film exposure. Molecular weight markers used were the See blue plus 2 prestained standards (Invitrogen).

### 5-Azacytidine treatment

LNCaP cells were cultured in RPMI-1640 medium as described above for 2 days in 12-well plates. A final concentration of 1 or 5 *μ*M of freshly prepared 5-azacytidine (Sigma-Aldrich) was added to the culture medium. After 5 days of treatment, RNA was isolated using TRI reagent as described above.

### Bisulphite modified DNA sequencing and primer design

The methylation status of the S100A6 gene (promoter region and 5′ upstream region of exon 1) was investigated in the S100A6 negative cell lines and in the Du145 cell line, by sequencing of bisulphite modified DNA (Herman *et al*, 1996). To design sequencing primers an appropriate area of the S100A6 promoter and exon 1 was identified using the published human S100A6 sequence (Gene bank accession number; J02763), and the MethPrimer design programme (http://itsa.ucsf.edu/~urolab/m ethprimer/). The bisulphite modified sense primer sequence was (IRD 800 labelled) 5′ CTCCCCCTATCTCCTCTAAAATAAC 3′, which corresponds to position −1084 to −1109 (designating the ATG of the translational start site as 0). The bisulphite modified antisense primer was (IRD 700 labelled) 5′ TGGGGGAGTTTAGTAGTGTTTATTG 3′, which corresponds to position −842 to −867. This 267 bp fragment encompassed the promoter region and the first 41 nucleotides 5′ of exon 1. Genomic DNA was extracted by a standard phenol–chloroform method. In total, 1 *μ*g of DNA was treated with sodium bisulphite for 16 h, using the CpGenome kit (Serologicals, UK), according to the manufacturer's instructions. After purification, a 2 *μ*l aliquot was used for sequencing using the EXCEL II sequencing kit-LC (Epicentre technologies), according to the manufacturer's instructions. Sequencing was performed in both the forward and reverse direction. The sequencing PCR products were resolved on an automated sequencer (LICOR) and aligned using the Sequencher programme.

## RESULTS

### S100A6 staining in benign prostate tissue

**Figure 1:**
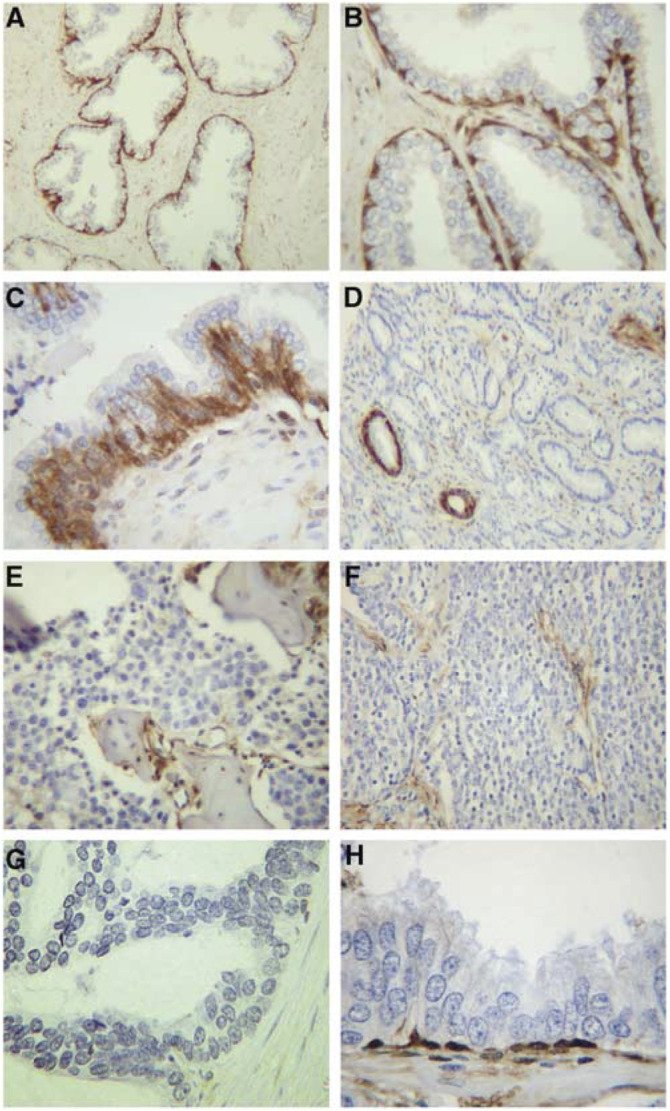
S100A6 immunostaining in benign, malignant, metastatic prostate and high-grade PIN samples. (**A**) Note the intense staining of the basal cells within the epithelium, seen as a single continuously stained cell layer (× 200 magnification). (**B**) Higher magnification shows intense staining in the cytoplasm and nuclei of basal cells but an absence of staining in luminal cells (× 400). (**C**) Staining within an area of basal cell proliferation (× 400). Note the intense staining in the hyperproliferative basal cells, with the luminal cells showing absent staining. (**D**) Adenocarcinoma showing absent S100A6 expression within the malignant cells, but intense expression in basal cells of benign glands (× 200). Absent staining of metastatic cells was seen in (**E**) bone and (**F**) lymph node metastasis (× 400). (**G**) A case of high-grade PIN with a disrupted basal cell layer showing absent staining (× 400). (**H**) A case of high-grade PIN with an intact basal cell layer, showing intense S100A6 staining within the basal cells (× 400).

Immunostaining for S100A6 showed that in all 66 cases of benign epithelium adjacent to prostatic adenocarcinoma, the staining was seen to be intense in the nuclei and cytoplasm of basal cells, with luminal cells showing an absence of staining. Overall, the staining pattern was seen as a single layer of stained cells within the typical two-layered architecture (Figure 1A and B

). Many of the specimens in blocks showed foci of basal cell proliferations. These are benign lesions where the basal cells have proliferated to become multilayered. S100A6 immunostaining showed intense staining in all basal cell layers with the single layer of luminal cells showing an absence of staining (Figure 1C). In all cases absent or weak-moderate staining was seen in the smooth muscle cells within the benign stroma (Figure 1A and B).

### S100A6 staining in prostate cancer and metastatic lesions

In contrast to the intense expression seen in the basal cell layer of benign glands, a complete loss of S100A6 staining was seen in all 66 cases of adenocarcinomas, regardless of Gleason score (Figure 1D). Absent staining was seen in all five cases of metastatic cancer (Figure 1E and F).

### S100A6 staining in high-grade prostatic intraepithelial neoplasia

In 10 cases of adenocarcinoma, we examined 10 lesions of high-grade prostatic intraepithelial neoplasia (PIN) chosen at random. High-grade PIN lesions consist of architecturally benign prostatic acini lined by cytologically atypical cells in which the basal cell layer may show some disruption (Bostwick *et al*, 1997). S100A6 immunostaining was found to be absent in three cases of PIN where the basal cell layer was disrupted (Figure 1G, and was found to be present in the basal cells of the seven cases of high-grade PIN where an intact basal cell layer was present (Figure 1H).

### Comparison of S100A6 staining with cytokeratin 5 and 18

**Figure 2:**
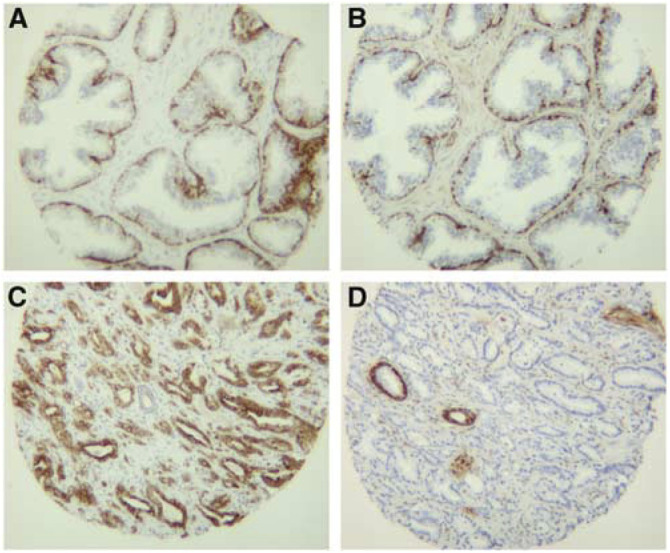
Comparison of S100A6 immunostaining with cytokeratin 5 and cytokeratin 18 immunostaining (× 100 magnification). (**A**) and (**B**) are serial sections of benign tissue stained with (**A**) cytokeratin 5 or (**B**) S100A6. Note that the staining patterns are similar with intense staining of the basal cell layer. In (**B**), note the weak-moderate staining of the muscle cells within the stroma. (**C**) and (**D**) are serial sections of adenocarcinoma stained with (**C**) cytokeratin 18 or (**D**) S100A6. Note that the cytokeratin 18 positive cells are not stained for S100A6. Basal cells of benign glands adjacent to carcinoma are stained for S100A6 (**D**).

To compare the S100A6 staining pattern with that of cytokeratin 5 and cytokeratin 18, tissues arranged in a TMA, comprising of 19 cases of matched adenocarcinoma, benign epithelium and benign stroma were serially cut and immunostained for cytokeratin 5 (a marker of prostate basal cells), or cytokeratin 18 (a marker for benign luminal cells), which is also expressed in malignant cells (Freeman *et al*, 2002). As expected, in all cases of benign epithelium intense cytokeratin 5 immunostaining was seen in the basal cell layer, with an absence of staining in the luminal cells (Figure 2A

), and in all cases this staining pattern was identical to the staining pattern seen with S100A6 (Figure 2B). In cases where weak-moderate S100A6 staining was seen in the smooth muscle cells of the stroma, this was not seen with cytokeratin 5 (Figures 2A and B). As expected, intense cytokeratin 18 immunostaining was seen in the luminal cells of benign epithelium and malignant cells (Figure 2C). Staining of serial sections showed that the benign luminal cells and malignant cells stained with cytokeratin 18 were unstained for S100A6 (Figures 2C and D).

To confirm the S100A6 immunostaining pattern seen using the polyclonal anti-S100A6 antibody (Dako), we immunostained serial sections of tissues in blocks and in the TMA, with a mouse monoclonal anti-S100A6 antibody (Sigma). Concordant staining patterns were seen in all cases. However, some variation in the intensity of staining of the stromal compartment was seen between the two antibodies, with the monoclonal antibody showing slightly less staining of the stromal compartment (data not shown).

### S100A6 expression in prostate cancer cell lines

**Figure 3:**
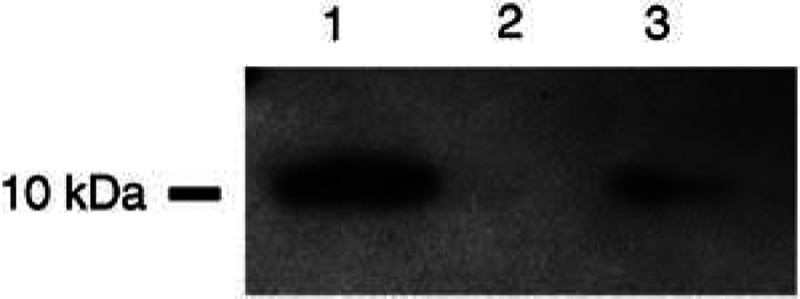
Western blot of S100A6, using the polyclonal anti-S100A6 antibody (Dako) on lysates from prostate cancer cell lines. Lane 1. Du145; lane 2, LNCaP; lane 3, PC3. Note the protein expression in Du145 and PC3 cells, seen as an ∼10 kDa band, but its absence in the LNCaP cell line.

S100A6 expression was investigated by Western blotting in all seven cell lines using the anti-S100A6 polyclonal antibody. Protein expression was seen in Du145, PC3, PC-3M, PC-3M-LN4 cell lines as a single ∼10 kDa band, thus confirming the specificity of the antibody used. However, protein expression was absent in the LNCaP, LNCaP-LN3, LNCaP-Pro5 cell lines. Figure 3

shows Western blotting data from representative cell lines (Du145, LNCaP and PC3). These data were confirmed using two different protein extraction protocols. To further confirm the expression of S100A6 in cell lines, we performed immunocytochemistry on Du145, PC3 and LNCaP cell lines. Cytoplasmic protein expression was seen in both Du145 and PC3 cells with weak expression in the nucleus (data not shown). LNCaP cells as expected showed a complete absence of S100A6 expression.

**Figure 4:**
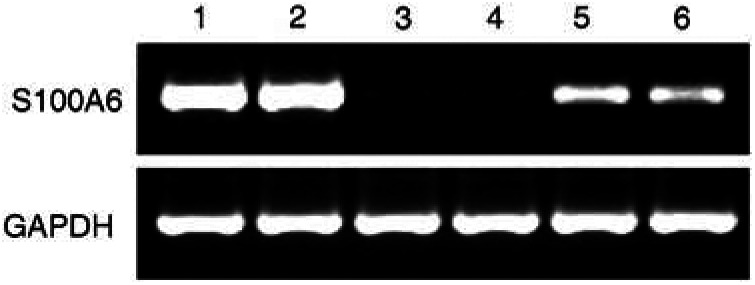
RT–PCR of S100A6 using the S100A6 F2/R2 primers ran on a 2.5% agarose/ethidium bromide gel. S100A6 mRNA expression can be seen in lane 1 (Du145), lane 2 (PC3), but not in lane 3 (LNCaP) or lane 4 (using a two-fold excess of LNCaP cDNA). Treatment of LNCaP cells with 5-Azacytidine caused re-expression of S100A6 mRNA using two different doses, lane 5 (1 *μ*M) and lane 6 (5 *μ*M). Nontranscribed total RNA or water used as negative controls showed an absence of PCR product (not shown). GAPDH was used as a loading control in each case and to check RNA integrity.

To confirm the protein expression status in the cell lines, we performed RT–PCR using two separate PCR primer pairs (S100A6 F1/R1 and S100A6 F2/R2), on RNA extracted from all seven cell lines. Using both primer sets, S100A6 mRNA expression was detected in Du145, PC3, PC-3M, PC-3M-LN4 cells, as seen by the correct sized PCR products, but was absent/very weak in the LNCaP, LNCaP-LN3 and LNCaP-Pro5 cells. Results using the S100A6 F2/R2 primer pair for representative cell lines (Du145, LNCaP and PC3) are shown (Figure 4

), but identical results were seen using the S100A6 F1/R1 primer pair (not shown).

### S100B is not expressed in prostate

In neuronal cell lines it has been shown that S100B may form heterodimers with S100A6 and that S100B protein expression is co-localised with S100A6 (Deloulme *et al*, 2000), we therefore performed immunostaining for S100B in 20 cases of organ confined prostatic adenocarcinoma with adjacent benign epithelium. S100B expression was completely absent in all cases of benign and malignant tissues. Melanoma samples included as positive controls showed intense S100B expression within the malignant cells (data not shown).

### S100A6 mRNA is re-expressed following 5-azacytidine treatment

Previous studies have shown an inverse correlation between expression of S100A6 and methylation in human keratinocytes and fibroblasts (Elder and Zhao, 2002), we therefore investigated whether this could be the mechanism of silencing in LNCaP cells. Cells were treated with 5-Azacytidine for 5 days using two doses: 1 and 5 *μ*M. S100A6 mRNA expression was reactivated in LNCaP cells following treatment with both doses and was seen using both primer sets. Data using primer pair S100A6F2/R2 are shown (Figure 4, lanes 5 and 6).

### CpG sites within the S100A6 promoter and exon 1 are methylated in prostate cancer cell lines

**Figure 5:**
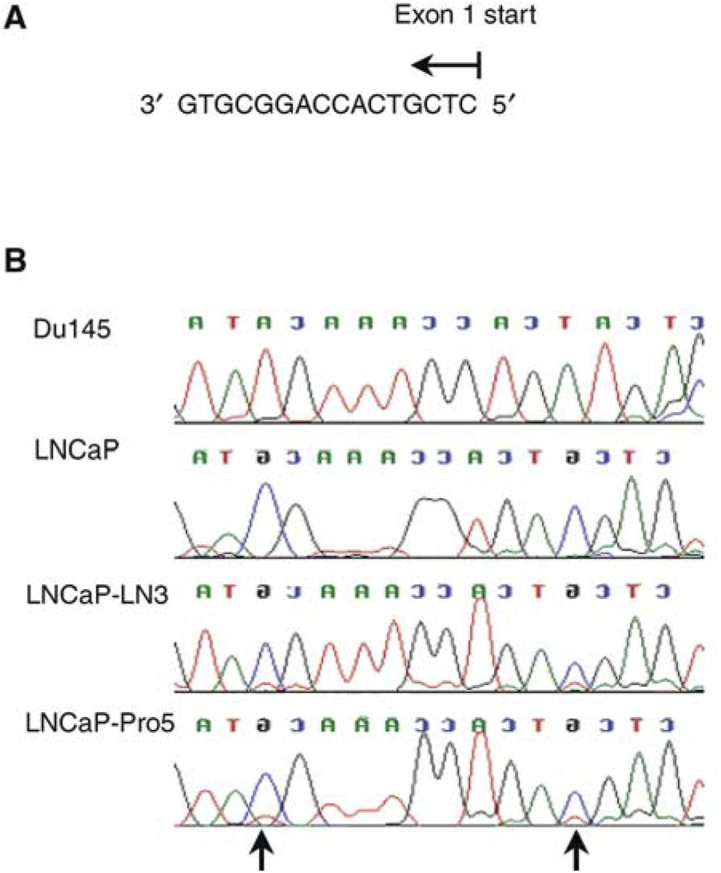
S100A6 DNA sequence of prostate cancer cell lines. (**A**) Sequence of the 5′ upstream region of exon 1, which was investigated (Gene bank Accession number J02763). The two CpG sites are shown in bold (**B**) Chromatographs showing S100A6 exon 1 sequence following bisulphite modification of DNA from prostate cancer cell lines. Du145 showed an absence of methylation as seen by the conversion of all the G residues to A residues. Methylation at the two CpG sites (arrows) can be seen in the LNCaP, LNCaP-LN3 and LNCaP-Pro5 cell lines, since the G residues at these sites were not converted, but remained as G residues.

In view of the above results, we hypothesised that CpG methylation may underlie the absence of S100A6 protein expression in the LNCaP, LNCaP-LN3 and LNCaP-Pro5 cell lines. Bisulphite modified DNA sequencing of a region of the S100A6 promoter and 5′ upstream region of exon 1 in LNCaP, LNCaP-LN3 and LNCaP-Pro5 cell lines identified sites of CpG methylation, which were unmethylated in the Du145 cell line (Figure 5

).

## DISCUSSION

Previous studies have shown that loss of Annexins and GST-pi are among the most common alterations occurring in prostate cancer, with the frequency of loss approaching 100% (Lee *et al*, 1994; Paweletz *et al*, 2000; Chetcuti *et al*, 2001). This mechanism of loss is thought to involve hypermethylation, suggesting that epigenetic mechanisms may play a significant role in the development of prostate cancer.

Annexins belong to one of the three main family of calcium-binding proteins. The other family of calcium binding proteins are the EF type and include the S100 proteins, such as S100B and S100A6 (Schafer and Heizmann, 1996; Heizmann *et al*, 2002; Donato, 2003). The third family comprises the endoplasmic reticulum proteins, such as calreticulin (Michalak *et al*, 2002). Evidence exists for interactions between two different family members such as Annexin II and S100A6 and between S100B and S100A6 (Zeng *et al*, 1993; Kuo *et al*, 1997; Deloulme *et al*, 2000).

The human S100A6 gene is located on chromosome 1q21, and the protein product has been implicated in growth and differentiation as well as in a variety of human cancers (Calabretta *et al*, 1986; Weterman *et al*, 1992; Weterman *et al*, 1993). To our knowledge there is only one recent report investigating the tissue expression of S100A6 in prostatic adenocarcinoma, which showed that ∼20% of the cases arranged on a TMA had S100A6 expression (Hsieh *et al*, 2003). However, the limitations of this study are that no cases of benign tissues were studied and the distribution of staining in different cell types was not described, that is, no distinction between luminal or basal cells was made. Given the results of previous studies showing S100A6 expression to be increased in all cancers as well as the correlation between expression and malignant transformation and invasion, our finding of loss of S100A6 expression in all adenocarcinomas studied was unexpected. The frequency of S100A6 loss we observed in our series of adenocarcinomas is comparable to the frequency of loss reported for Annexin I and II. Thus, loss of expression of proteins belonging to the calcium-binding protein family is among the most frequent events in prostate cancer development.

The finding of S100A6 loss of expression in a significant proportion of high-grade PIN lesions as well as in all cases of adenocarcinoma, regardless of Gleason score and in metastatic lesions, suggests that loss of expression occurs at an early stage of prostate tumour development and that loss of expression is maintained throughout tumour progression. In addition, this loss of expression was related to the loss of the basal cell phenotype in high-grade PIN and cancer.

Analysis of human prostate cancer cell lines showed expression of S100A6 protein and mRNA in Du145 (derived from metastatic prostate in the brain), PC3 (derived from metastatic prostate in the bone), PC-3M and PC-3M-LN4 (metastatic variants of PC3), whereas the LNCaP cell line (derived from a lymph node metastasis) and LNCaP-LN3 and LNCaP-Pro5 cell lines (metastatic variants of LNCaP) (Pettaway *et al*, 1996) showed an absence of protein and mRNA. Although, the absence of S100A6 expression seen in all tumours was confirmed in the LNCaP cell line and its variants, other metastatic cell lines such as Du145 and PC3 (and its variants), were shown to express S100A6. The reason(s) for the differences in S100A6 expression seen between the human metastatic tissues and certain cell lines is not known, but it is possible that re-expression could have occurred at some point during the establishing of the cell lines. Nevertheless, the finding of S100A6 expression in some prostate cancer cell lines but not others provides a useful tool to further understand the mechanism(s) involved in regulating S100A6 expression.

The pattern of S100A6 immunostaining seen in benign prostate epithelium was similar to the pattern seen for cytokeratin 5 and that reported for p63 and 34*β*E12 (a cocktail of cytokeratins), which are markers of prostate basal cells (Parsons *et al*, 2001; Freeman *et al*, 2002). In all cases of adenocarcinoma studied, loss of expression of both S100A6 and cytokeratin 5 was seen in the malignant cells. This frequency of loss is similar to the frequency of loss (∼95%) reported for p63 in prostate cancer (Parsons *et al*, 2001). Therefore, S100A6 immunohistochemistry could represent a novel marker for basal cells in the benign prostate and its absence in malignant cells may be useful as an adjuvant method to p63 and 34*β*E12 immunostaining to facilitate the pathological diagnosis of prostate cancer.

The loss of expression seen in the malignant cells was confirmed using two commercially available antibodies, that is, a mouse monoclonal antibody (Sigma) and rabbit polyclonal antibody (Dako). Both antibodies showed identical staining patterns, using serial sections, although the monoclonal antibody showed slightly reduced staining in the stromal compartment. The S100A6 polyclonal antibody was shown to be specific for S100A6, by the finding of a single band of the correct molecular size (∼10 kDa) in Du145, PC3, PC-3M and PC-3M-LN4 cell lines. In addition, the absence of protein in LNCaP cells was very unlikely to be due to proteolytic cleavage since no low molecular weight bands were seen on the Western blots.

Since yeast two-hybrid experiments have shown S100A6 to interact with S100B and co-localise in astrocytoma cells (Deloulme *et al*, 2000), we investigated the expression of both S100A6 and S100B in serial sections of prostatic adenocarcinoma of various Gleason scores and adjacent benign epithelium. We found no evidence of S100B protein expression in the prostate whereas intense expression was seen in the malignant melanoma cases used as positive controls (data not shown). This suggests that in contrast to previous studies, S100A6 is not co-expressed with S100B and that S100B is unlikely to play a role in the normal function of S100A6 in the prostate.

As the mechanism of S100A6 loss of expression has been correlated with DNA methylation in human keratinocytes and fibroblasts (Elder and Zhao, 2002), we investigated the mechanism of S100A6 silencing in the LNCaP cell line using the drug 5-Azacytidine. 5-Azacytidine is a DNA methylation inhibitor that effectively inactivates the DNA methyltransferase enzyme. Several *in vitro* and *in vivo* studies have reported that 5-Azacytidine can cause hypomethylation and reverse the epigenetic silencing of tumour suppressor genes in tumour cell lines (Bender *et al*, 1998; Bovenzi *et al*, 1999). Our finding that S100A6 mRNA could be re-activated following treatment of cells with 5-Azacytidine suggested that hypermethylation could be the mechanism for silencing of the S100A6 gene. However, more significantly sequencing of bisulphite modified DNA in LNCaP, LNCaP-LN3 and LNCaP-Pro5 cell lines showed that CpG methylation was present, but was not present in Du145 cells which showed high expression of S100A6 protein.

In summary, we have shown intense S100A6 expression in the basal cells of benign prostate epithelium. Loss of expression was seen in high-grade PIN, all grades of adenocarcinoma, all metastatic prostate lesions and in the LNCaP prostate cancer derived cell line. The high frequency of loss of expression parallels the frequency of loss seen in other calcium-binding proteins such as Annexin I and II. The pattern of S100A6 expression in benign epithelium and adenocarcinomas is similar to that of cytokeratin 5 and that reported for p63 and 34*β*E12, and therefore has the potential to be used as an adjuvant to these markers in the diagnosis of prostate cancer. The mechanism underlying the loss of expression appears to involve methylation of CpG sites within the promoter region and exon 1. Further studies regarding the expression of S100A6 in prostate cancer and other tumours are clearly warranted.
